# Prevalence of salivary gland hypertrophy syndrome in laboratory colonies and wild flies of *Glossina pallidipes* in Ethiopia

**DOI:** 10.4102/ojvr.v82i1.896

**Published:** 2015-06-01

**Authors:** Mahder M. Yimer, Dereje G. Bula, Tsegabirhan K. Tesama, Kassaw A. Tadesse, Birhanu H. Abera

**Affiliations:** 1 College of Veterinary Medicine, Mekelle University, Ethiopia; 2 Kaliti Tsetse Mass Rearing and Irradiation Centre, Southern Tsetse Fly Eradication Project, Ministry of Science and Technology, Addis Ababa, Ethiopia

## Abstract

*Glossina pallidipes* salivary gland hyperplasia (GpSGH) syndrome caused by the salivary gland hyperplasia virus reduces the reproduction potential of tsetse flies, posing a serious threat for rearing of sufficient colonies for use of tsetse and trypanosome control using the sterile insect technique. This research was conducted in the Kaliti Tsetse Mass Rearing and Irradiation Centre in Ethiopia with the objective of studying the prevalence of GpSGH syndrome in laboratory colonies of *G. pallidipes* (Tororo and Arbaminch) reared for release in the implementation of the sterile insect technique and a field strain of *G. pallidipes* Arbaminch. Presence or absence of GpSGH was determined when pathological features of the salivary gland were revealed after dissection. The overall prevalence of GpSGH syndrome in laboratory colonies was 48.3% (747/1548) with a statistically significant (*z *=* *17.30, *p *= 0.001) prevalence of 70.2% (544/775) in Arbaminch colonies and 26.26% (203/773) in Tororo colonies. The prevalence of GpSGH in laboratory flies fed according to the clean blood feeding protocol was 68.9% and 22.4% in Arbaminch and Tororo strains respectively. It was 70.5% and 27.2% respectively in laboratory colonies of Arbaminch and Tororo strains fed according to the standard membrane feeding protocol. The difference in prevalence of the disease between the two feeding protocols was not statistically significant in either Arbaminch (*z* = 0.361, *p* = 0.359) or Tororo (*z* = 1.22, *p* = 0.111) strains. The prevalence of SGH in wild *G. pallidipes* Arbaminch strain was 3% (15/500) and was significantly (*z* = 23.61, *p* < 0.001) lower than in the laboratory strain. The effect of age and density-related stress on the development of GpSGH was not statistically significant. The prevalence of GpSGH in the newly emerging (teneral) flies in the laboratory colonies was 66.7% and 20% in the Arbaminch and Tororo strains respectively. For all considered risk factors, the prevalence was much higher in *G. pallidipes* Arbaminch laboratory colonies.

## Introduction

The debilitating diseases African sleeping sickness in humans, caused by *Trypanosoma brucei gambiense* and *T. b. rhodesiense* and African animal trypanosomosis (AAT), caused by *T. b. brucei*, *T. congolense* and *T. vivax*, are cyclically transmitted by tsetse flies (*Glossina* species) (Simarro, Louis & Jannin 2003; World Health Organization 2003). In Ethiopia, an area of about 220 000 km^2^ is infested by *G. morsitans submorsitans*, *G. pallidipes*, *G. tachinoides*, *G. fuscipes fuscipes* and *G. longipennis*, contributing as biological vectors of *T. congolense*, *T. vivax* and *T. brucei* (Abebe 2005). Tesfaye *et al*. (2012) reported that trypanosomosis is responsible for substantial economic losses through cattle mortality, cost incurred for drug purchase and other control interventions and loss of draft power of infected oxen. On the other hand, Shaw *et al*. (2014) indicated huge economic advantages that can be accessed from implementing tsetse and trypanosomosis control interventions in currently infected areas.

Control of tsetse flies and use of trypanocidal drugs for treatment or prophylaxis are the two control options for trypanosomosis; however, both have many drawbacks, such as the high cost of purchase, environmental pollution by insecticides, increasing trypanocidal drug resistance and lack of new drugs to replace them (Chaka & Abebe 2003). Vector control through the sterile insect technique (SIT) is an alternative strategy for the sustainable management of these diseases (Leak 1998). Successful eradication of *G. austeni* on the island of Unguja, Republic of Tanzania, entailed an area-wide integrated pest management approach (Hendrichs *et al*. 2007) that included the release of sterile male flies (Vreysen *et al*. 2000), and serves as a starting point for the wider recommendation of the technique in Africa. In 1996, the government of Ethiopia, in collaboration with the United Nations’ International Atomic Energy Agency (IAEA), initiated a tsetse eradication project called the Southern Rift Valley Tsetse Eradication Project (STEP) using SIT (Alemu *et al*. 2007; Feldmann *et al*. 2005).

SIT requires production of surplus males that are later gamma sterilised for field release and maintenance of females breeding for colony turnover. Failure to raise big colonies as a result of the presence of *G. pallidipes* salivary gland hypertrophy/hyperplasia virus (GpSGHV) is the main stumbling block for SIT application and could detrimentally affect tsetse control, with consequent serious health and economic implications (Abd-Alla *et al*. 2010; Sasanya *et al*. 2010). The virus replicates in the nucleus of salivary gland cells, causing distinct tissue hypertrophy in the female milk gland and male gonadal tissues, resulting in ovarian abnormalities and testicular degeneration, thus reducing the reproductive potential in both sexes of *G. pallidipes* (Sang *et al*. 1999). Vertical transmission of GpSGHV from mother to offspring either by trans-ovarian transfer of virus to embryo (Jura, Otieno & Chimtawi 1989) or via virus-infected milk glands to developing larvae (Sang *et al*. 1999) are the principal transmission routes. In laboratory-maintained flies, horizontal transmission during *in vitro* feeding of blood provided under a silicone membrane (Feldmann 1994) was believed to be a major route of virus infection, which is enhanced as a result of confinement in rearing cages (Malele *et al*. 2013).

The presence of *G. pallidipes* salivary gland hypertrophy/hyperplasia (GpSGH) in colonies at Kaliti Tsetse Mass Rearing and Irradiation Centre (TMRIC) and Arbaminch wild colonies was reported by Abd-Alla *et al*. (2010), constituting a potential challenge to maintaining sufficient laboratory-reared tsetse fly colonies to be irradiated and released. Determining the magnitude and associated risk factors of the disease could contribute to designing applicable control options for the virus infection in tsetse flies. Therefore, this study was designed to determine the prevalence of GpSGH syndrome in two laboratory colonies of *G. pallidipes* (Tororo and Arbaminch) and a wild strain of *G. pallidipes* Arbaminch.

## Materials and methods

### Description of the study areas

This study was conducted from November 2011 to June 2012. The laboratory work was conducted in the TMRIC, Kaliti, Addis Ababa, located 8°50′N to 9°06′N and 38°05′E to 39°05′E (Addis Ababa City Administration, 1998, cited by Nigatu, Rajan & Bizunesh 2011). Wild *G. pallidipes* Arbaminch strains were collected at selected sampling points in the district of Arbaminch in the Gamo Goffa Zone of the Southern Nations, Nationalities, and Peoples’ Region (SNNPR), about 500 km south of Addis Ababa, located between 4°45′N and 7°15′N and 36°40′E and 35°20′E. The climate varies between humid and windy, with annual precipitation between 300 mm and 700 mm in the lowlands and between 500 mm and 1200 mm in the highlands. The average temperature ranges between 11 °C and 38 °C (Alemu *et al*. 2007).

### Tsetse fly populations

Tsetse flies from two laboratory colonies of *G. pallidipes* were used in this study, (1) a colony maintained and originating from pupae collected near Tororo, Uganda (Tororo colony) and (2) a colony established at the TMRIC from pupae collected near Arbaminch (Arbaminch colony) since 1996. To check the condition of GpSGH syndrome in the field populations, wild *G. pallidipes* of the Arbaminch strain were collected from the Arbaminch area. Collection and sex identification based on hypopygium structure were carried out as described previously (FAO 1992). The flies were handled in standard cages and transported to the TMRIC. These flies were dissected immediately on arrival; no feeding and mixing with laboratory colonies was allowed. Age was not determined in these flies.

### Blood feeding protocols

Two protocols for feeding the flies were used, (1) the standard membrane feeding protocol, whereby successive cages with flies were offered a blood meal on the same membrane (Feldmann 1994) and (2) a clean blood feeding protocol, whereby each cage of flies was provided with new, sterile defibrinated bovine blood at each meal, which prevents the flies from picking up the virus from the blood used for feeding previous cages (Abd-Alla *et al*. 2010). Both feeding systems were evaluated for their effect on the prevalence of GpSGH. The clean blood feeding protocol was conducted in seven cages of each laboratory strain, containing 30 male flies per cage, at each feeding time. These flies were progeny of parent stocks that were fed under the standard membrane feeding protocol. In the clean feeding protocol, newly-emerged (teneral) *G. pallidipes* flies of both strains were offered a clean blood meal and thereafter were always fed on fresh, clean blood during the entire period of the experiment (Table 1). The tsetse colonies used to study the effect of density-related stress (Table 2) and age (Table 3) were fed using the standard membrane feeding protocol.

**TABLE 1:** Prevalence of *Glossina pallidipes* salivary gland hyperplasia in *Glossina pallidipes* Arbaminch and *Glossina pallidipes* Tororo laboratory colonies (clean membrane feeding).

Feeding time	*Glossina pallidipes* Arbaminch		*Glossina pallidipes* Tororo	
	Dissected	Positive	%	Dissected	Positive	%
Teneral (unfed)	30	20	66.7	30	6	20.0
Week 3	28	19	67.9	28	6	21.4
Week 6	29	20	69.0	27	6	22.2
Week 9	26	18	69.2	30	7	23.3
Week 12	27	19	70.4	21	5	23.8
Week 15	21	15	71.4	16	4	25.0
**Total**	**161**	**111**	**68.9**	**152**	**34**	**22.4**

**TABLE 2:** Effect of stress (density) on prevalence of *Glossina pallidipes* salivary gland hyperplasia in *Glossina pallidipes* Arbaminch and *Glossina pallidipes* Tororo laboratory colonies (standard membrane feeding).

Density	*Glossina pallidipes* Arbaminch					*Glossina pallidipes* Tororo					
	Dissected	Positive	%	Odds ratio	*p*-value	Density	Dissected	Positive	%	Odds ratio	*p*-value
38	32	22	68.8	-	-	38	31	7	22.6	-	***-***
68	55	38	69.1	1.17	0.739	68	50	13	26.0	1.20	0.729
120	92	64	69.6	1.20	0.679	120	80	21	26.3	1.22	0.690
**226**	**179**	**124**	**69.3**	**-**	**-**	**-**	**161**	**41**	**21.5**	**-**	**-**

**TABLE 3:** Effect of age on the prevalence of *Glossina pallidipes* salivary gland hyperplasia in *Glossina pallidipes* Arbaminch and *Glossina pallidipes* Tororo laboratory –colonies (standard membrane feeding).

Age	*Glossina pallidipes* Arbaminch colony					*Glossina pallidipes* Tororo colony				
	Dissected	Positive	%	Odds ratio	*p*-value	Dissected	Positive	%	Odds ratio	*p*-value
Day 10	88	60	68.2	-	-	89	21	23.5	-	-
Week 2	29	20	69.0	1.04	0.937	25	6	24.0	1.02	0.966
Week 3	17	12	70.6	1.12	0.845	30	8	26.7	1.18	0.735
Week 4	20	14	70.0	1.09	0.874	26	7	26.9	1.36	0.527
Week 5	27	19	70.4	1.10	0.830	29	8	27.6	1.23	0.665
Week 6	24	17	70.8	1.13	0.804	25	7	28.0	1.26	0.652
Week 7	28	20	71.4	1.17	0.746	27	8	29.6	1.36	0.527
Week 8	24	17	70.8	1.13	0.804	30	9	30.0	1.39	0.486
Week 9	25	18	72.0	1.20	0.716	28	8	28.6	1.30	0.595
Week 10	29	21	72.4	1.23	0.669	24	7	29.2	1.33	0.576
Week 11	22	16	72.7	1.24	0.680	17	5	29.4	1.35	0.610
Week 12	26	19	73.1	1.27	0.635	26	8	30.8	1.44	0.460
Week 13	15	11	73.3	1.28	0.691	23	7	30.4	1.42	0.501
Week 14	19	14	73.7	1.31	0.638	26	8	30.8	1.44	0.460
Week 15	23	17	73.9	1.32	0.596	16	5	31.3	1.47	0.515
Week 16	19	14	73.7	1.3	0.638	19	6	31.6	1.49	0.468
**Total**	**435**	**309**	**71.0**	**-**	**-**	**460**	**128**	**27.8**	**-**	**-**

#### Effect of stress (density) on the prevalence of GpSGH syndrome

Male teneral flies were selected randomly from both strains of the *G. pallidipes* colonies and maintained in standard colony holding cages (20 cm × 20 cm × 5 cm) at densities of 38, 68 and 120 flies per cage (Table 2), as indicated by Abd-Alla *et al*. (2010). Each cage was maintained at 24 °C and 70% – 80% humidity for 20 days. After 20 days, all the tsetse flies were dissected and the condition of the salivary glands was recorded.

#### Effect of age on the prevalence of GpSGH syndrome

Tsetse flies in the Kaliti TMRIC live for a maximum period of 17 weeks (4 months). A total of 17 cages of different age groups of both strains were selected, each containing 30 male flies kept on a regimen of clean membrane blood feeding three times per week. Age was determined from standard records available in the centre. From each age group, a minimum of 15 tsetse flies of both strains (one cage of each) were dissected at the age of 10 days and at weekly intervals from week 2–16 (Table 3).

### Tsetse fly dissection procedures and identification of post-mortem GpSGHV lesions

Tsetse flies were dissected under a stereo microscope according to the procedures indicated by the FAO (1992). Immediately afterwards the glands were mounted in 5% saline and examined under the microscope. Pathological features of salivary gland hypertrophy/hyperplasia virus (SGHV) described by Gouteux (1987) and Verena *et al*. (2011) were used to distinguish between infected and non-infected flies.

Briefly, on dissection of the tsetse flies, normal salivary glands were transparent and the diameter of the distal part of the gland was about 100 µm, with almost no variation throughout the life of the tsetse fly, whilst hypertrophied glands were enlarged (diameter could reach up to 1.1 mm more than their normal thickness) and chalky-white or bluish in appearance.

### Statistical analysis

Statistical Package for Social Sciences (SPSS) version 17 statistical software (SPSS 2007) was used for data entry and analysis. Comparison of the prevalence (%) of GpSGH was made by logistic regression. Odds ratio was used to report the effect of the discrete study variables (stress and age) on the development of GpSGH syndrome. The *Z*-test for comparison of two population proportions was used to check the statistical significance of the prevalence of GpSGH in the strains, prevalence related to standard membrane and clean blood feeding protocols of the laboratory colonies and wild tsetse fly strains from the Arbaminch area. In all analyses, the confidence level was held at 95% (Thrusfield 2005).

## Results

A total of 2048 tsetse flies (1548 laboratory adapted and 500 wild) were dissected. The overall prevalence of GpSGH syndrome in laboratory colonies was 48.3% (747/1548) with a statistically significant (*z* = 17.30, *p* < 0.001) prevalence of 70.2% (544/775) in Arbaminch and 26.3% (203/773) in Tororo colonies (Tables 1–3). The prevalence of GpSGH in laboratory flies fed using the clean blood feeding protocol was 68.9% (111/161) and 22.4% (34/152) in Arbaminch and Tororo strains respectively (Table 1). Likewise, the prevalence in laboratory colonies fed using the standard membrane feeding protocol was 70.5% (433/614) and 27.2% (169/621) in Arbaminch and Tororo strains respectively (Tables 2 & 3). This variation in prevalence of the disease with both feeding protocols was not statistically significant in either Arbaminch (*z* = 0.361, *p* = 0.359) or Tororo (*z* = 1.22, *p* = 0.111) strains. The prevalence of GpSGH in the wild *G. pallidipes* Arbaminch strain was 3% (15/500). A statistically non-significant (*z* = 1.31, *p* = 0.095) difference in prevalence of 2.5% (10/400) and 5% (5/100) in female and male tsetse flies respectively was observed. The prevalence of GpSGH in the *G. pallidipes* Arbaminch laboratory colony (70.2%) was significantly higher than in the wild G*. pallidipes* Arbaminch strain (3%) (*z* = 23.61, *p* < 0.001).

In flies fed using the standard membrane feeding protocol, there was a statistically non-significant increase in prevalence of GpSGH syndrome from 68.2% to 73.7% in Arbaminch laboratory colonies and from 23.7% to 31.6% in Tororo colonies as the age of tsetse flies increased (Table 3). Moreover, as the feeding time on the clean membrane increased, there was a statistically non-significant increase in the prevalence of GpSGH syndrome from 66.7% to 71.4% in Arbaminch colonies and from 20.0% to 25.0% in Tororo colonies. The prevalence of GpSGH in the newly emerging (teneral) flies was 66.7% and 20% in Arbaminch and Tororo strains respectively (Table 1).

As the density of tsetse flies within a cage increased, there was no difference in the prevalence of GpSGH syndrome in either *G. pallidipes* Arbaminch laboratory colonies or *G. pallidipes* Tororo colonies (Table 2).

## Discussion

Monitoring the prevalence of GpSGH through dissections is an important component of quality control (Abd-Alla *et al*. 2010; Abd-Alla *et al*. 2013a). The prevalence of GpSGH in Arbaminch laboratory colonies fed using standard membrane feeding was 70.5%, which was comparable to results of Abd-Alla *et al*. (2013b), who indicated a prevalence of 73.84% on dissection of old Kaliti Arbaminch colonies. The prevalence of GpSGH in Tororo colonies fed using the standard membrane feeding was 27.2%. Abd-Alla *et al*. (2010) had reported on the prevalence of GpSGH from 2007–2009 in Arbaminch (22.4%, 43.5%, 27% respectively) and Tororo (≤ 10%) colonies in the Kaliti TMRIC. The clean feeding regimen decreased the virus copy number (Abd-Alla *et al*. 2010; Lietze *et al*. 2009) and is the recommended feeding strategy to reduce the virus load and thus the prevalence of SGH syndrome, according to Abd-Alla *et al*. (2013b). However, even though some of the tsetse flies dealt within our study (Table 1) were fed using this feeding regimen, the prevalence of the disease was nevertheless high and not statistically different from the prevalence in the flies fed using the standard membrane. The prevalence of GpSGH in Arbaminch strains was significantly higher (*p* < 0.001) than in Tororo strains.

The high prevalence of GpSGH syndrome in the teneral and adult laboratory colonies fed using the clean membrane protocol (Table 1) and the notable proportion in the wild strains could be explained by an infection acquired from the parent stocks. Vertical transmission of SGHV from mother to offspring either by trans-ovarian transfer of virus to embryo (Jura *et al*. 1989) or via virus-infected milk glands to developing larvae (Sang *et al*. 1999) has been documented as the principal transmission route. However, the role of horizontal transmission in maintaining and disseminating the infection should not be underestimated, as the virus is continuously shed in the saliva of infected flies (Abd-Alla *et al*. 2007). The feeding habit of tsetse flies – emptying saliva content prior to feeding into the medium (blood meal) – causes virus particle accumulation at each bite, which facilitates the exposure of the healthy susceptible colony (Abd-Alla *et al*. 2011). Abd-Alla *et al*. (2010, 2013a) reported that attempts to use the membrane feeding technique to up-scale production in a *G. pallidipes* facility in Ethiopia was extremely challenging because of rapid spread of GpSGHV in the colonies. The absence of a statistically insignificant increase in the prevalence of SGH in *G. pallidipes* Arbaminch and *G. pallidipes* Tororo laboratory colonies (Table 2) as the density increased was in agreement with Abd-Alla *et al*. (2010). Moreover, as the age of the flies increased (Table 3), a statistically non-significant increase in the prevalence of SGH was observed. This could indicate the absence of immune-based resistance in teneral and adult flies and stress factors such as density and age might not play a significant role in disease transmission dynamics.

For all the study variables considered, the prevalence of GpSGH syndrome in *G. pallidipes* Tororo strain was significantly lower than in *G. pallidipes* Arbaminch strain. This observation was in agreement with Abd-Alla *et al*. (2011, 2013a). According to Abd-Alla *et al*. (2010), this low prevalence in Tororo strains could be as a result of (1) lower pathogenicity of this virus compared to that of the Ethiopian strain, (2) an acquired resistance of the flies in this colony to the virus or (3) a different virus copy number in asymptomatic and symptomatic flies, or a combination of the three.

The prevalence of GpSGH in the wild Arbaminch flies in our study (3%) was comparable to the reports of Abd-Alla *et al*. (2013b) in the new Arbaminch laboratory colony (4.47%). However, this figure should not be underestimated. According to Kariithi *et al*. (2013) and Abd-Alla *et al*. (2007), an asymptomatic course of the virus infection might have contributed to the low prevalence of GpSGH in wild tsetse flies. Using molecular diagnostic tools, Kariithi *et al*. (2013) reported a prevalence of 68.9% in Arbaminch wild tsetse populations, in which mother-to-offspring transmission, either trans-ovum or through infected milk glands, is thought to be the most likely mode of transmission of the virus (Jura *et al*. 1989; Sang *et al*. 1998).

Successful implementation of an area-wide integrated pest management programme with SIT requires large-scale production and release of sterile males of acceptable quality, that is, males capable of competing with wild males for mating with wild tsetse females (Hendrichs *et al*. 2007). However, infections with SGHV markedly reduce the already low natural reproductive potential of tsetse flies kept for breeding purposes. Infected female flies have reduced reproductive organs and male *G. pallidipes* positive to SGHV infection are unable to inseminate female flies; releasing those infected sterile flies will reduce the efficiency of a sterile male release programme (Gouteux 1987; Mutika *et al*. 2012). To alleviate the heavy economic losses associated with GpSGHV and ensure successful SIT programmes, various control and prevention strategies are envisaged. Prior to incorporating them in the breeding colonies, parent colonies have to be screened with appropriate diagnostic tools, and a combination of antiviral drug treatment and using the clean feeding system could aid better health management of the breeding colonies (Abd-Alla *et al*. 2011, 2014).

In conclusion, high infection rates in these colonies warrant replacing the existing breeding colonies with new, healthy ones. Because of the presence of the infection in wild colonies, the selection of new breeding colonies from wild populations has to be based on careful screening with appropriate diagnostic tools. Improved health management strategies coupled with other husbandry practices could contribute to better management of new breeding colonies. Further studies to identify comparative roles of vertical and horizontal transmission cycles on the disease dynamics could help in designing alternative control strategies.

## References

[CIT0001] Abd-Alla A.M.M., Bergoin M., Parker A.G., Maniania N.K., Vlak J.M., Bourtzis K. et al, 2013a, ‘Improving sterile insect technique (SIT) for tsetse flies through research on their symbionts and pathogens’, *Journal of Invertebrate Pathology* 112 Suppl, S2–S10. http://dx.doi.org/10.1016/j.jip.2012.07.009 22841636 10.1016/j.jip.2012.07.009 PMC4242710

[CIT0002] Abd-Alla A.M.M., Bossin H., Cousserans F., Parker A., Bergoin M. & Robinson A., 2007, ‘Development of a non-destructive PCR method for detection of the salivary gland hypertrophy virus (SGHV) in tsetse flies’, *Journal of Virological Methods* 139(2), 143–149. http://dx.doi.org/10.1016/j.jviromet.2006.09.018 17070938 10.1016/j.jviromet.2006.09.018

[CIT0003] Abd-Alla A.M.M., Kariithi H.M, Andrew G.P., Robinson A.S., Kiflom M., Bergoin M. et al, 2010, ‘Dynamics of the salivary gland hypertrophy virus in laboratory colonies of *Glossina pallidipes* (Diptera: Glossinidae)’, *Viruses* 150, 103–110. http://dx.doi.org/10.1016/j.virusres.2010.03.001 10.1016/j.virusres.2010.03.001 20214934

[CIT0004] Abd-Alla A.M.M., Kariithi H.M., Mohamed A.H., Lapiz E., Parker A.G. & Vreysen M.J.B., 2013b, ‘Managing hytrosavirus infections in *Glossina pallidipes* colonies: Feeding regime affects the prevalence of salivary gland hypertrophy syndrome’, PLoS ONE 8(5), e61875 http://dx.doi.org/10.1371/journal.pone.0061875 23667448 10.1371/journal.pone.0061875 PMC3646844

[CIT0005] Abd-Alla A.M.M., Marin C., Parker A.G. & Vreysen M.J.B., 2014, ‘Antiviral drug valacyclovir treatment combined with a clean feeding system enhances the suppression of salivary gland hypertrophy in laboratory colonies of *Glossina pallidipes*’, *Parasites and Vectors* 7(214), 1–4. http://dx.doi.org/10.1186/1756-3305-7-214 24886248 10.1186/1756-3305-7-214 PMC4026819

[CIT0006] Abd-Alla A.M.M., Salem T.Z., Parke A.G., Wang Y., Jehle J.A., Vreysen M.J.B. et al, 2011, ‘Universal primers for rapid detection of hytrosaviruses’, *Journal of Virological Methods* 171, 280–283. http://dx.doi.org/10.1016/j.jviromet.2010.09.025 20923688 10.1016/j.jviromet.2010.09.025

[CIT0007] Abebe G., 2005, ‘Trypanosomosis in Ethiopia’, *Ethiopian Journal of Biological Sciences* 4(1), 75–121.

[CIT0008] Alemu T., Kapitano B., Mekonnen S., Aboset G. & Kiflom M., 2007, ‘Area wide control of tsetse and trypanosomosis: Ethiopian experience in the Southern Rift Valley’, in Vreysen M.J.B, Robinson A.S. & Hendrichs J.(Eds.) , *Area-wide control of insect pests: From research to field implementation*, pp. 325–335, Springer, Dordrecht http://dx.doi.org/10.1007/978-1-4020-6059-5_30

[CIT0009] Chaka H. & Abebe G., 2003, ‘Drug resistant trypanosomes: A threat to cattle production in the south west of Ethiopia’, *Revue d'Elevage et de Médecine Vétérinaire des Pays Tropicaux* 56(1–2), 33–36.

[CIT0010] FAO, 1992, *Training manual for tsetse control personnel*, Vol. 1, viewed 15 March 2015, from http://www.fao.org/docrep/009/p5178e/P5178E00.htm#TOC

[CIT0011] Feldmann U., 1994, ‘Guidelines for the rearing of tsetse flies using the membrane feeding technique’, in Ochieng’-Odero J.P.R. , *Techniques of insect rearing for the development of integrated pest and vector management strategies*, pp. 449–471, ICIPE Science Press, Nairobi.

[CIT0012] Feldmann U., Dyck V.A., Mattioli R.C. & Jannin J., 2005, ‘Potential impact of tsetse fly control involving the sterile insect technique’, in Dyck V.A., Hendrichs J. & Robinson A.S.(Eds.) , *Sterile insect technique: Principles and practice in area-wide integrated pest management*, pp. 701–723, Springer, Dordrecht http://dx.doi.org/10.1007/1-4020-4051-2_27

[CIT0013] Gouteux J.P., 1987, ‘Prevalence of enlarged salivary glands in *Glossina palpalis, G. pallicera*, and *G. nigrofusca* (Diptera: Glossinidae) from the Vavoua area, Ivory Coast’, *Journal of Medical Entomology* 24(2), 268 http://dx.doi.org/10.1093/jmedent/24.2.268 3035182 10.1093/jmedent/24.2.268

[CIT0014] Hendrichs J.P., Kenmore P., Robinson A.S. & Vreysen M.J.B., 2007, ‘Area-wide integrated pest management (AW-IPM): Principles practice and prospects’, in Vreysen M.J.B., Robinson A.S. & Hendrichs J.(Eds.) , *Area-wide control of insect pests: From research to field implementation*, pp. 3–33, Springer, Dordrecht.

[CIT0015] Jura W.G.Z.O., Otieno L.H. & Chimtawi M.M.B., 1989, ‘Ultrastructural evidence for trans-ovum transmission of the DNA virus of tsetse *Glossina pallidipes* (Diptera: Glossinidae)’, *Current Microbiology* 18, 1–4. http://dx.doi.org/10.1007/BF01568821

[CIT0016] Kariithi H.M., Ahmadi M., Parker A.G., Franz G., Ros V.I.D, Haq I. et al, 2013, ‘Prevalence and genetic variation of salivary gland hypertrophy virus in wild populations of the tsetse fly *Glossina pallidipes* from southern and eastern Africa’, *Journal of Invertebrate Pathology* 112 (Suppl)., S123–S132. http://dx.doi.org/10.1016/j.jip.2012.04.016 22634094 10.1016/j.jip.2012.04.016

[CIT0017] Leak S.G.A., 1998, *Tsetse biology and ecology: Their role in the epidemiology and control of trypanosomosis*, CABI Publishing, Wallingford.

[CIT0018] Lietze V.U., Sims K.R., Salem T.Z., Geden C.J. & Boucias D.G., 2009, ‘Transmission of MdSGHV among adult house flies, *Musca domestica* (Diptera: Muscidae), occurs via oral secretions and excreta’, *Journal of Invertebrate Pathology* 101(1), 1–7. http://dx.doi.org/10.1016/j.jip.2009.02.007 19254721 10.1016/j.jip.2009.02.007

[CIT0019] Malele I.I., Manangwa O., Nyingilili H.H., Kitwika W.A, Lyaruu E.A., Msangi A.R. et al, 2013, ‘Prevalence of SGHV among tsetse species of economic importance in Tanzania and their implication for SIT application’, *Journal of Invertebrate Pathology* 112 Suppl, S133–S137. http://dx.doi.org/10.1016/j.jip.2012.07.018 22841949 10.1016/j.jip.2012.07.018

[CIT0020] Mutika G.N., Marin C., Parker A.G., Boucias D.G., Vreysen M.J.B. & Abd-Alla A.M.M., 2012, ‘Impact of salivary gland hypertrophy virus infection on the mating success of male *Glossina pallidipes*: Consequences for the sterile insect technique’, *PLoS One* 7(8), e42188 http://dx.doi.org/10.1371/journal.pone.0042188 22912687 10.1371/journal.pone.0042188 PMC3418267

[CIT0021] Nigatu R., Rajan D.S. & Bizunesh B.S., 2011, ‘Challenges and opportunities in municipal solid waste management: The case of Addis Ababa city, central Ethiopia’, *Journal of Human Ecology* 33(3), 179–190.

[CIT0022] Sang R.C., Jura W.G.Z.O, Otieno L.H. & Mwangi R.W., 1998, ‘The effects of a DNA virus infection on the reproductive potential of female tsetse flies, *Glossina morsitans centralis* and *Glossina morsitans morsitans* (Diptera: Glossinidae)’, *Memórias do Instituto Oswaldo Cruz* 93, 861–864. http://dx.doi.org/10.1590/S0074-02761998000600030 9921317 10.1590/s0074-02761998000600030

[CIT0023] Sang R.C., Jura W.G.Z.O., Otieno L.H., Mwangi R.W. & Ogaja P., 1999, ‘The effects of a tsetse DNA virus infection on the functions of the male accessory reproductive gland in the host fly *Glossina morsitans centralis* (Diptera: Glossinidae)’, *Current Microbiology* 38(6), 349–354. http://dx.doi.org/10.1007/PL00006815 10341076 10.1007/pl00006815

[CIT0024] Sasanya J.J., Abd-Alla A.M.M., Parker A.G. & Cannavana A., 2010, ‘Analysis of the antiviral drugs acyclovir and valacyclovir-hydrochloride in tsetse flies (*Glossina pallidipes*) using LC–MSMS’, *Journal of Chromatography B* 878(26), 2384–2390. http://dx.doi.org/10.1016/j.jchromb.2010.07.008 10.1016/j.jchromb.2010.07.008 20719583

[CIT0025] Shaw A.P., Cecchi G., Wint G.R., Mattioli R.C. & Robinson T.P., 2014, ‘Mapping the economic benefits to livestock keepers from intervening against bovine trypanosomosis in Eastern Africa’, *Preventive Veterinary Med*icine 113(2), 197–210. http://dx.doi.org/10.1016/j.prevetmed.2013.10.024 24275205 10.1016/j.prevetmed.2013.10.024

[CIT0026] Simarro P.P., Louis F.J. & Jannin J., 2003, ‘Sleeping sickness, forgotten illness: What are the consequences in the field?’, *Médecine Tropicale, Revue du Corps de Sante Colonial* 63(3), 231–235. 14579457

[CIT0027] SPSS, 2007, *SPSS Statistics Base 17.0 User's Guide*, 17th edn, SPSS Inc, Chicago.

[CIT0028] Tesfaye D., Speybroeck N., De Deken R. & Thys E., 2012, ‘Economic burden of bovine trypanosomosis in three villages of Metekel zone, northwest Ethiopia’, *Tropical Animal Health and Production* 44(4), 873–879. http://dx.doi.org/10.1007/s11250-011-9981-3 21935660 10.1007/s11250-011-9981-3

[CIT0029] Thrusfield M., 2005, *Veterinary epidemiology*, 2nd edn, Blackwell Science, London.

[CIT0030] Verena U.L., Abd-Alla A.M.M., Marc J.B.V., Christopher J.G. & Drion G.B., 2011, ‘Salivary gland hypertrophy viruses: A novel group of insect's pathogenic viruses’, *Annual Review of Entomology* 56, 63–80. http://dx.doi.org/10.1146/annurev-ento-120709-144841 10.1146/annurev-ento-120709-144841 20662722

[CIT0031] Vreysen M.J.B., Saleh K.M., Ali M.Y., Abdulla A.M. & Zhu Z.R., 2000, ‘*Glossina austeni* (Diptera: Glossinidae) eradicated on the island of Unguja, Zanzibar, using the sterile insect technique’, *Journal of Economic Entomology* 93(1), 123–135. http://dx.doi.org/10.1603/0022-0493-93.1.123 14658522 10.1603/0022-0493-93.1.123

[CIT0032] World Health Organization, 2003, *Report of the Scientific Working Group meeting on African trypanosomiasis*, 04–08 June, 2001, Geneva.

